# Overall survival following radical surgery and postoperative chemotherapy in colorectal cancer patients aged ≥ 70 years: a population-based retrospective cohort study

**DOI:** 10.1186/s12885-025-14863-w

**Published:** 2025-09-02

**Authors:** Ji-Yeon Mun, Kyungdo Han, Nina Yoo, Bong-Hyeon Kye, InKyu Lee

**Affiliations:** 1 Department of Surgery, The Catholic University of Korea, St. Vincent’s Hospital, Suwon, the Republic of Korea; 2 Department of Statistics and Actuarial Science, Soongsil University, Seoul, Republic of Korea; 3 The Catholic University of Korea, Seoul St. Mary’s Hospital, Seoul, the Republic of Korea

**Keywords:** Colorectal neoplasms, Frailty, Comorbidity, Drug therapy, Surgical procedures

## Abstract

**Background:**

Colorectal cancer (CRC) predominantly affects the elderly, with a growing demand for evidence-based treatment strategies in this population. The influence of comorbidities and frailty on survival outcomes following postoperative chemotherapy in elderly CRC patients remains unclear.

**Method:**

From the Korean National Health Insurance database, we analyzed 200,000 randomly sampled colorectal cancer patients (C18-20) diagnosed between 2009 and 2016. Patients under 20, those with inflammatory bowel disease, preoperative chemotherapy (except neoadjuvant chemoradiotherapy for rectal cancer), missing data, or death within 6 months post-surgery were excluded. Cox proportional hazards models estimated hazard ratios (HRs) for overall survival, adjusted for demographic factors. Subgroup and interaction analyses assessed effect modification by age, sex, and income. Incidence rates were reported per 1,000 person-years.

**Result:**

Among 2,142 patients, 44.2% received postoperative chemotherapy. Chemotherapy recipients were younger and had lower overall mortality in the unadjusted analysis. However, after adjustment, postoperative chemotherapy was associated with an increased risk of mortality (adjusted hazard ratio [HR] 1.44, 95% CI: 1.10–1.87) in patients aged ≥ 70 years. The impact of chemotherapy on survival was more pronounced in younger patients. Comorbidity and frailty emerged as significant prognostic factors, potentially outweighing the benefits of adjuvant therapy in the elderly.

**Conclusion:**

In elderly CRC patients, comorbidity and frailty may play a dominant role in determining overall survival, potentially exceeding the impact of tumor-directed treatments such as postoperative chemotherapy. These findings highlight the need for comprehensive geriatric assessment and individualized treatment planning in this population.

## Introduction

Colorectal cancer (CRC) is the third most commonly occurring cancer and the second most frequent cause of cancer death worldwide [1]. Globally, 60.4% of all cases were aged between 50 and 74 years at diagnosis [2]. Despite CRC in young individuals under 50 years is rapidly growing, the median age of onset is estimated as 67 years, and 80% of CRC occurs at the age of 70 and older [2]. As the elderly population expands, the demand for standard colorectal cancer treatments to older patients is on the rise.

Ultimately, the aim of therapy is tumor eradication and long-term remission. If not possible, at least the treatment focuses on maintaining stable disease status while preserving a patient’s functional capacity. This may include radical surgery, with chemoradiotherapy being incorporated before and after the surgery, depending on the disease status at the initial presentation. Expectedly, prognosis depends on the stage of disease with expected 5-year OS of 70% for patients with stage II disease and 45–65% for patients with stage III disease according to the American Joint Committee on Cancer [3, 4]. Furthermore, with advancement of treatment options for systemic and locoregional disease control, the life expectancy of patients with metastatic CRC has increased up to 32 to 40 months [4]. 

Previously, several studies have shown that comorbidities significantly influence the prognosis of patient survival [5–7]. In particular, concurrent comorbidity and frailty emerge as robust prognostic factors for survival in patients with CRC, beyond the commonly acknowledged sociodemographic and tumor characteristics [7]. As the aging population continues to grow, planning effective treatment for elderly patients becomes increasingly complex, given their frequent presentation with multiple comorbid conditions and heightened frailty. Therefore, it is essential to assess the effectiveness of current treatment strategies, which primarily include radical surgery and chemotherapy and/or radiotherapy, in elderly patients. This study investigates whether elderly patients (≥ 70 years) with CRC derive meaningful survival benefit from postoperative chemotherapy after radical resection using the National Sample Cohort (NSC) database from the Korean National Health Insurance Service (KNHIS).

## Materials and methods

### Study population and database

This is a nationwide, population-based, observational, retrospective cohort study. We utilized a sample cohort database provided by the KNHIS, which is composed of 2% patient samples that are randomly extracted from qualified National Health Insurance (NHI) subscribers and medical aid beneficiaries [8]. With nearly 97% of the Korean population enrolled, this national insurance service provides comprehensive medical coverage for both inpatient and outpatient care. The KNHIS has developed a database containing diagnoses (classified according to the International Classification of Diseases, 10th revision or ICD-10), procedures, prescription records, demographic information, and medical costs. The KNHIS manages and releases public data that includes demographic information, medical facility usage records, and diagnostic codes based on the Korean Standard Classification of Diseases, 7th Revision (KCD-7). The KCD-7 classification system aligns with the ICD-10 standards.

In this study, we analyzed patients diagnosed with CRC between 2009 and 2016. The exclusion criteria comprised individuals who had surgical treatment more than three months after their initial diagnosis, those with a prior diagnosis of ulcerative colitis or Crohn’s disease, and patients who received systemic chemotherapy before surgical intervention, with the exception of those who underwent concurrent chemoradiotherapy for rectal cancer. Additionally, patients who died within six months of diagnosis were also excluded.

This study complied with the principles outlined in the Declaration of Helsinki and received approval from the Institutional Review Board of the Catholic University of Korea, St. Vincent’s Hospital (IRB # KC23ZISI0642). All procedures performed in studies involving human participants were in accordance with the ethical standards of the institutional and/or national research committee and with the 1964 Helsinki Declaration and its later amendments or comparable ethical standards. Informed consent from individual patients was waived by the Institutional Review Board due to the retrospective design of the study, as the data utilized were public and anonymized in accordance with confidentiality guidelines.

### Definition of colorectal cancer, treatments, and confounders

CRC was identified based on the ICD-10 codes: colon cancer (C18 and C19) and rectal cancer (C20). Surgical treatment was defined by the procedural codes for colectomy, rectal and sigmoid colon resection, Hartmann’s procedure, and/or total or subtotal colectomy. We defined preoperative chemotherapy as any codes related to the mixing and infusion of chemotherapeutic agents, any substances related to irinotecan, capecitabine, or oxaliplatin, and/or nursing care for outpatient chemotherapy injections. Concurrent chemoradiotherapy was defined by codes that combine chemotherapy with substances related to 5-fluorouracil and radiation.

### Statistical analysis

Baseline characteristics according to the receipt of chemotherapy were compared using the Student’s t-test for continuous variables and the chi-squared test for categorical variables. All data are presented as mean ± standard deviation (SD). Cox proportional hazards regression analysis was performed to evaluate the association between chemotherapy and mortality among patients with colorectal cancer. Incidence rates were calculated per 1,000 person-years of follow-up, stratified by treatment status and age groups (< 70 and ≥ 70 years). Hazard ratios (HRs) with 95% confidence intervals (CIs) were estimated using Cox proportional hazards models to compare mortality risks between treated and untreated groups. To account for potential age-related differences in treatment effects, we conducted stratified analyses, adjusted for sex, age, and low income status.

To adjust for potential confounding factors, we constructed and compared three models independently. Model 1 was analyzed without adjustment. Model 2 adjusted for age, sex, cancer stage, and comorbidities including hypertension, diabetes mellitus, and dyslipidemia. Model 3 included additional adjustments for body mass index (BMI), smoking status, alcohol consumption, and socioeconomic status, based on the variables included in Model 2. Hazard ratios (HRs) and 95% confidence intervals (CIs) were calculated to assess the risk of mortality associated with chemotherapy.

To further investigate the influence of other factors on the association between chemotherapy and mortality, subgroup analyses were conducted. Subgroup analyses were performed according to age group (≥ 65 years), sex, cancer stage, and the presence of major comorbidities such as diabetes mellitus and hypertension. All statistical analyses were performed using SAS version 9.4 (SAS Institute Inc., Cary, NC, USA). A *p*-value of < 0.05 was considered statistically significant.

## Results

Table 1 summarizes patient demographics and characteristics. Of 2,142 patients, 44.2% received postoperative chemotherapy. Patients who received chemotherapy were significantly younger (mean age 60.7 vs. 67.0 years, *p* < 0.0001). Among those aged ≥ 70, a higher proportion of males received chemotherapy (63.6% vs. 53.6%, *p* = 0.0099). Colon cancer was more common in chemotherapy recipients under 70 (77.9% vs. 73.0%, *p* = 0.0398). Colectomy was more frequent in non-chemotherapy patients aged ≥ 70 (46.5% vs. 36.9%, *p* = 0.0119). Mortality was lower in the chemotherapy group overall (22.2% vs. 29.0%, *p* = 0.0003), especially in patients under 70 (7.9% vs. 25.8%, *p* < 0.0001), but not in those aged ≥ 70. No significant differences were observed in low-income status or cancer site distribution in the overall cohort.

**Table 1:** Patient demographics and characteristics

		Total	Postoperative chemotherapy (in Total)			Postoperative chemotherapy in Age<70 (*N*=1343)			Postoperative chemotherapy in Age≥70 (*N*=799)		
			No	Yes	*p*	No	Yes	*p*	No	Yes	*p*
*N* (%)		2142 (100)	1197 (55.8)	945 (44.2)		634 (47.2)	709 (52.8)		563 (70.4)	236 (29.9)	
Sex	Male	1273(59.43)	701(58.56)	572(60.53)	0.3575	399(62.93)	422(59.52)	0.2002	302(53.64)	150(63.56)	0.0099
	Female	869(40.57)	496(41.44)	373(39.47)		235(37.07)	287(40.48)		261(46.36)	86(36.44)	
Age	Mean±SD	64.24±12.02	67±12.01	60.74±11.09	<.0001	57.91±8.53	56.4±9.2	0.002	77.24±5.17	73.77±3.47	<.0001
	20-39	64(2.99)	24(2.01)	40(4.23)	<.0001	24(3.79)	40(5.64)	0.0093			<.0001
	40-59	659(30.77)	291(24.31)	368(38.94)		291(45.9)	368(51.9)				
	60-69	620(28.94)	319(26.65)	301(31.85)		319(50.32)	301(42.45)				
	70-79	602(28.1)	381(31.83)	221(23.39)					381(67.67)	221(93.64)	
	≥80	197(9.2)	182(15.2)	15(1.59)					182(32.33)	15(6.36)	
Social status	Low income status	354(16.53)	192(16.04)	162(17.14)	0.4951	111(17.51)	124(17.49)	0.9929	81(14.39)	38(16.1)	0.5346
Colorectal ICD-10 code											
Colon	C18-C19	1633(76.24)	899(75.1)	734(77.67)	0.1657	463(73.03)	552(77.86)	0.0398	436(77.44)	182(77.12)	0.9206
Rectum	C20	509(23.76)	298(24.9)	211(22.33)		171(26.97)	157(22.14)		127(22.56)	54(22.88)	
Type of surgery	Colectomy	808(37.72)	478(39.93)	330(34.92)	0.0586	216(34.07)	243(34.27)	0.991	262(46.54)	87(36.86)	0.0119
	Anterior resection or Low anterior resection	1330(62.09)	717(59.9)	613(64.87)		416(65.62)	464(65.44)		301(53.46)	149(63.14)	
	Total or subtotal colectomy	4(0.19)	2(0.17)	2(0.21)		2(0.32)	2(0.28)				
Time from surgery to first chemo (days)		96.05±265.12	820.73±602.41	37±21.83	<.0001	839.49±613.35	35.49±19.86	<.0001	803.38±599.41	41.54±26.43	<.0001
Death		540(25.21)	266(22.22)	274(28.99)	0.0003	50(7.89)	183(25.81)	<.0001	216(38.37)	91(38.56)	0.9591
Follow-up duration	Mean±SD	5.37±2.69	5.53±2.67	5.16±2.71	0.0013	6.26±2.39	5.35±2.67	<.0001	4.72±2.75	4.57±2.73	0.4921
	Median (Q1-Q3)	5.31 (3.35-7.46)	5.6 (3.52-7.61)	4.96 (3.18-7.23)	0.0035	6.32 (4.38-8.18)	5.22 (3.45-7.42)	<.0001	4.56 (2.67-6.88)	4.23 (2.57-6.71)	0.452

		Total	Postoperative chemotherapy (in Total)			Postoperative chemotherapy in Age<70 (*N*=1343)			Postoperative chemotherapy in Age≥70 (*N*=799)		
			No	Yes	*p*	No	Yes	*p*	No	Yes	*p*
*N* (%)		2142 (100)	1197 (55.8)	945 (44.2)		634 (47.2)	709 (52.8)		563 (70.4)	236 (29.9)	
Sex	Male	1273(59.43)	701(58.56)	572(60.53)	0.3575	399(62.93)	422(59.52)	0.2002	302(53.64)	150(63.56)	0.0099
	Female	869(40.57)	496(41.44)	373(39.47)		235(37.07)	287(40.48)		261(46.36)	86(36.44)	
Age	Mean±SD	64.24±12.02	67±12.01	60.74±11.09	<.0001	57.91±8.53	56.4±9.2	0.002	77.24±5.17	73.77±3.47	<.0001
	20-39	64(2.99)	24(2.01)	40(4.23)	<.0001	24(3.79)	40(5.64)	0.0093			<.0001
	40-59	659(30.77)	291(24.31)	368(38.94)		291(45.9)	368(51.9)				
	60-69	620(28.94)	319(26.65)	301(31.85)		319(50.32)	301(42.45)				
	70-79	602(28.1)	381(31.83)	221(23.39)					381(67.67)	221(93.64)	
	≥80	197(9.2)	182(15.2)	15(1.59)					182(32.33)	15(6.36)	
Social status	Low income status	354(16.53)	192(16.04)	162(17.14)	0.4951	111(17.51)	124(17.49)	0.9929	81(14.39)	38(16.1)	0.5346
Colorectal ICD-10 code											
Colon	C18-C19	1633(76.24)	899(75.1)	734(77.67)	0.1657	463(73.03)	552(77.86)	0.0398	436(77.44)	182(77.12)	0.9206
Rectum	C20	509(23.76)	298(24.9)	211(22.33)		171(26.97)	157(22.14)		127(22.56)	54(22.88)	
Type of surgery	Colectomy	808(37.72)	478(39.93)	330(34.92)	0.0586	216(34.07)	243(34.27)	0.991	262(46.54)	87(36.86)	0.0119
	Anterior resection or Low anterior resection	1330(62.09)	717(59.9)	613(64.87)		416(65.62)	464(65.44)		301(53.46)	149(63.14)	
	Total or subtotal colectomy	4(0.19)	2(0.17)	2(0.21)		2(0.32)	2(0.28)				
Time from surgery to first chemo (days)		96.05±265.12	820.73±602.41	37±21.83	<.0001	839.49±613.35	35.49±19.86	<.0001	803.38±599.41	41.54±26.43	<.0001
Death		540(25.21)	266(22.22)	274(28.99)	0.0003	50(7.89)	183(25.81)	<.0001	216(38.37)	91(38.56)	0.9591
Follow-up duration	Mean±SD	5.37±2.69	5.53±2.67	5.16±2.71	0.0013	6.26±2.39	5.35±2.67	<.0001	4.72±2.75	4.57±2.73	0.4921
	Median (Q1-Q3)	5.31 (3.35-7.46)	5.6 (3.52-7.61)	4.96 (3.18-7.23)	0.0035	6.32 (4.38-8.18)	5.22 (3.45-7.42)	<.0001	4.56 (2.67-6.88)	4.23 (2.57-6.71)	0.452

**Fig. 1:**
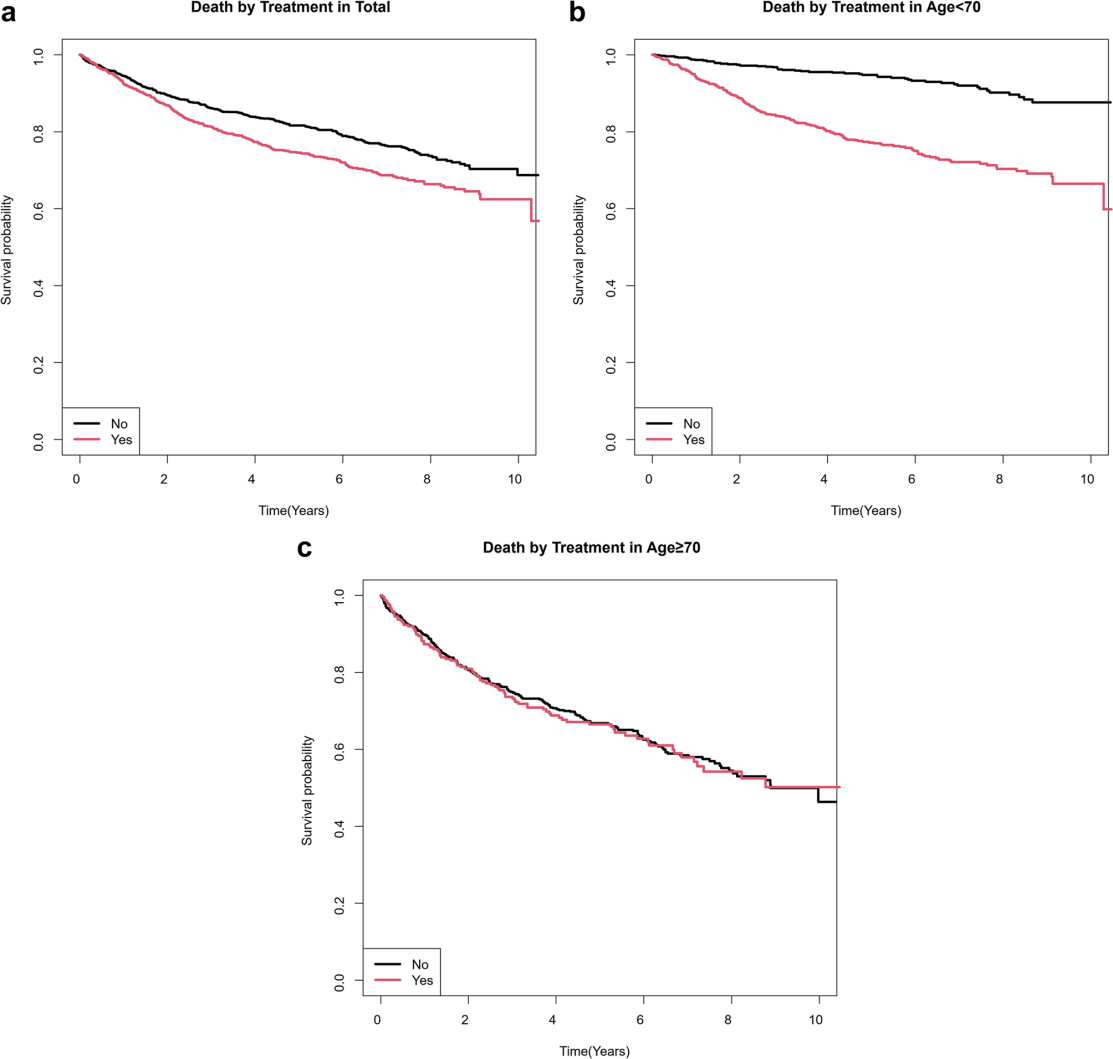
Kaplan-Meier survival curves of the patients who received postoperative chemotherapy compared to those who did not, (**a**) both in the overall cohort (**b**) patients younger than 70 years (**c**) aged 70 years or older

As shown in Fig. 1. Kaplan-Meier survival analysis demonstrated that patients who received postoperative chemotherapy had lower survival probabilities compared to those who did not receive chemotherapy. This pattern was observed in the overall cohort (Fig. 1a), as well as in both age subgroups: patients younger than 70 years (Fig. 1b) and those aged 70 years or older (Fig. 1c). In all groups, the survival curve for the chemotherapy group consistently lay below that of the non-chemotherapy group, indicating poorer survival among patients who underwent postoperative chemotherapy.

Table 2 presents the mortality risks associated with postoperative chemotherapy compared to no chemotherapy, with analyses stratified by age groups (< 70 and ≥ 70 years) and adjusted for relevant covariates. Overall, patients who received postoperative chemotherapy exhibited a higher incidence rate (IR) of mortality (74.1 per 1,000 person-years) compared to those who did not receive chemotherapy (66.6 per 1,000 person-years). The adjusted hazard ratio (HR) for mortality in the chemotherapy group was 1.877 (95% CI: 1.572–2.241), indicating a significantly increased risk compared to the non-chemotherapy group. In patients younger than 70 years, the adjusted HR was even higher at 3.831 (95% CI: 2.799–5.242), while in those aged 70 years or older, the adjusted HR was 1.437 (95% CI: 1.102–1.874).

**Table 2:** The mortality risks between postoperative chemotherapy and no chemotherapy groups

Postoperative chemotherapy		N	DEATH	Duration, PY	IR, 1000 PY	HR (95% CI)			
						Model 1	*P*	Model 2	*P*
in Total	No	1197	266	6624.61	40.15	1 (Ref.)	0.0002	1 (Ref.)	< 0.0001
	Yes	945	274	4874.09	56.22	1.382 (1.167,1.636)		1.877 (1.572,2.241)	
in Age < 70	No	634	50	3966.75	12.60	1 (Ref.)	< 0.0001	1 (Ref.)	< 0.0001
	Yes	709	183	3794.43	48.23	3.762 (2.751,5.146)		3.831 (2.799,5.242)	
in Age ≥ 70	No	563	216	2657.86	81.27	1 (Ref.)	0.8218	1 (Ref.)	0.0075
	Yes	236	91	1079.66	84.29	1.029 (0.805,1.314)		1.437 (1.102,1.874)	

Model 1 Non-adjusted

Model 2 Adjusted by sex, age, and income status

*PY* person-years, *IR *Incidence rate, *HR *Hazard ratio, *CI *Confidence interval

Table 3 shows that postoperative chemotherapy is associated with an increased risk of mortality after colorectal cancer treatment across most subgroups, with higher adjusted hazard ratios (HRs) seen especially in patients under 70 years old and in females. The increased risk is consistent regardless of sex or income status, though the magnitude varies. In patients aged 70 or older, the association between chemotherapy and mortality is weaker and not always statistically significant. Overall, the mortality risk linked to postoperative chemotherapy is most pronounced in younger and female patients.

**Table 3:** Subgroup analysis on the mortality risks after colorectal cancer treatment

		Postoperative chemotherapy	*N*	DEATH (*N*)	Duration, PY	IR, 1000-PY	HR (95% CI)			
							Model 1	*P* for interaction	Model 2	*P* for interaction
in Total										
Sex	Male	No	701	167	3878.43	43.06	1(Ref.)	0.8864	1(Ref.)	0.1333
		Yes	572	173	2891.66	59.83	1.367(1.105,1.69)		1.705(1.373,2.118)	
	Female	No	496	99	2746.18	36.05	1(Ref.)		1(Ref.)	
		Yes	373	101	1982.42	50.95	1.402(1.062,1.85)		2.231(1.675,2.972)	
Age	20–69	No	634	50	3966.75	12.60	1(Ref.)	< 0.0001	1(Ref.)	< 0.0001
		Yes	709	183	3794.43	48.23	3.75(2.742,5.127)		3.926(2.87,5.37)	
	≥ 70	No	563	216	2657.86	81.27	1(Ref.)		1(Ref.)	
		Yes	236	91	1079.66	84.29	1.031(0.807,1.317)		1.117(0.87,1.434)	
Income status	Others	No	1005	223	5610.62	39.75	1(Ref.)	0.6032	1(Ref.)	0.4921
		Yes	783	230	4045.94	56.85	1.409(1.172,1.694)		1.927(1.59,2.334)	
	Low	No	192	43	1013.99	42.41	1(Ref.)		1(Ref.)	
		Yes	162	44	828.14	53.13	1.248(0.82,1.899)		1.64(1.074,2.505)	
in Age < 70										
Sex	Male	No	399	40	2483.66	16.11	1(Ref.)	0.0649	1(Ref.)	0.0562
		Yes	422	114	2210.33	51.58	3.137(2.188,4.499)		3.135(2.186,4.496)	
	Female	No	235	10	1483.09	6.74	1(Ref.)		1(Ref.)	
		Yes	287	69	1584.1	43.56	6.387(3.29,12.4)		6.553(3.368,12.748)	
Income status	Others	No	523	38	3315.49	11.46	1(Ref.)	0.0631	1(Ref.)	0.0728
		Yes	585	157	3116.36	50.38	4.311(3.024,6.147)		4.368(3.062,6.232)	
	Low	No	111	12	651.26	18.43	1(Ref.)		1(Ref.)	
		Yes	124	26	678.07	38.34	2.076(1.048,4.115)		2.156(1.087,4.277)	
in Age ≥ 70										
Sex	Male	No	302	127	1394.77	91.05	1(Ref.)	0.5126	1(Ref.)	0.3184
		Yes	150	59	681.34	86.59	0.948(0.696,1.292)		1.313(0.951,1.813)	
	Female	No	261	89	1263.09	70.46	1(Ref.)		1(Ref.)	
		Yes	86	32	398.32	80.34	1.124(0.75,1.684)		1.702(1.119,2.589)	
Income status	Others	No	482	185	2295.13	80.61	1(Ref.)	0.307	1(Ref.)	0.2333
		Yes	198	73	929.58	78.53	0.97(0.74,1.272)		1.348(1.01,1.799)	
	Low	No	81	31	362.73	85.46	1(Ref.)		1(Ref.)	
		Yes	38	18	150.07	119.94	1.355(0.758,2.423)		1.992(1.1,3.609)	

Model 1 Non-adjusted

Model 2 adjusted by sex, Age, Income status

*PY* person-years, *IR *Incidence rate, *HR *Hazard ratio, *CI *Confidence interval

## Discussion

This nationwide, population-based cohort study investigating survival outcomes after postoperative chemotherapy following radical surgery for colorectal cancer demonstrates that survival was significantly worse in patients who received postoperative chemotherapy compared to those who did not, with an adjusted hazard ratio (HR) for mortality of 1.88 overall. The risk was notably higher in patients under 70 years old (adjusted HR 3.83), while it remained elevated but less pronounced in those aged 70 or older (adjusted HR 1.44). Among patients aged 70 or older, the association between chemotherapy and mortality was weaker and not always statistically significant. Overall, the negative impact on survival associated with postoperative chemotherapy was most pronounced in younger patients. Our findings suggest that in elderly patients with colorectal cancer, the impact of postoperative chemotherapy on overall survival is attenuated compared to younger patients. This may indicate that, in the elderly population, age-related factors—such as comorbidity burden, frailty, and limited life expectancy—may play a more decisive role in determining overall survival than tumor-directed interventions alone. In contrast, among younger patients, tumor-related factors and treatment effects may exert a greater influence on survival outcomes. This phenomenon is consistent with a previous finding indicating that the magnitude of benefit from adjuvant chemotherapy is lower in elderly patients compared to younger patients; specifically, the benefit is significantly greater in those under 70 years of age than in those aged 70 or older [9, 10]. 

The prognostic importance of comorbid conditions is most evident when the cancer itself is less aggressive and associated with better expected survival [5, 11]. In contrast, when the tumor is advanced or rapidly progressive, the influence of comorbidity on overall prognosis would be less significant. A large cohort study demonstrated that the effect of comorbidity on overall survival was most pronounced among patients with cancers associated with a relatively favorable prognosis—such as localized colon cancer—while it was less significant in those with advanced or aggressive cancers [6]. This evidence helps explain our observation that, in elderly patients with colorectal cancer, non-tumor factors, such as comorbidity and frailty, may play a dominant role in determining overall survival—potentially exceeding the impact of tumor-directed treatments like chemotherapy. As the burden of comorbidity increases with age, its influence on survival becomes more pronounced, especially in early-stage disease, and may mask or outweigh any benefits from adjuvant therapies [12]. 

Furthermore, a recent systematic review and meta-analysis evaluating the prognostic significance of frailty in patients with colorectal cancer demonstrated frailty was a strong predictor of both 30-day and 5-year mortality, with relative risks (RR) for mortality ranging from 2.26 to over 6.0 compared to non-frail patients [13]. This evidence reinforces our observation that age-related factors such as frailty and comorbidity may play a decisive role in survival among elderly patients with colorectal cancer, potentially outweighing the impact of tumor-directed treatments like chemotherapy. The authors further insisted that the importance of incorporating frailty assessment into clinical decision-making for elderly CRC patients, as frail individuals are at substantially higher risk for adverse outcomes regardless of the oncologic intervention. These may explain no clear benefit of adding oxaliplatin to 5-fluorouracil in adjuvant setting [14, 15]. 

This study has several limitations. Firstly, while our study examines associations between postoperative chemotherapy and overall survival across different age groups, we did not directly quantify or compare the relative impact of tumor-specific factors, such as stage or grade, versus patient-specific factors, such as comorbidity burden or age, on survival outcomes. As a result, our conclusions regarding the predominance of non-tumor factors in elderly patients are inferential, rather than directly demonstrated by our data. Secondly, our analysis did not include stratified survival curves or hazard models adjusted for tumor stage or comorbidity indices. Stage-specific survival analysis would allow for a clearer understanding of how the effect of chemotherapy varies within each stage, while comorbidity-adjusted analyses would help isolate the impact of treatment from underlying health status. The lack of these adjustments limits our ability to distinguish whether observed survival differences are primarily driven by cancer progression or by competing risks from other illnesses, particularly in the elderly cohort. Finally, there is a possibility of selection bias in our study population, especially among elderly patients. In clinical practice, patients with significant frailty, multiple comorbidities, or poor performance status are less likely to be offered or to tolerate postoperative chemotherapy. Conversely, those elderly patients who do receive chemotherapy may represent a selectively healthier subgroup, or, alternatively, may be those with more aggressive disease. This confounding by indication could distort the observed association between chemotherapy and survival, making it difficult to determine whether poorer outcomes are due to the treatment itself, the underlying health of the patient, or the biology of more advanced tumors. Without detailed data on performance status, frailty measures, or reasons for treatment selection, this potential bias cannot be fully addressed.

## Conclusion

This study suggests that, in elderly patients with colorectal cancer, non-tumor factors of comorbidity and frailty may play a dominant role in determining overall survival—potentially exceeding the impact of tumor-directed treatments like surgery and chemotherapy. As the burden of comorbidity frailty increases with age, its influence on survival becomes more pronounced, especially in early-stage disease, and may mask or outweigh any benefits from adjuvant therapies. However, these findings should be interpreted with caution due to several important limitations. Future research incorporating more detailed clinical data and robust analytical methods is needed to clarify the interplay between tumor characteristics, patient comorbidities, and treatment effects in elderly cancer patients.

## Data Availability

The datasets generated during and/or analyzed during the current study are available from the corresponding author on reasonable request.

## Abbreviations

CRC: Colorectal cancer
OS: Overall survival
HR: Hazard ratio
CI: Confidence interval
NHI: National health insurance
AJCC: American Joint Committee on Cancer
TNM: Tumor, node, metastasis (cancer staging system)
IBD: Inflammatory Bowel Disease
CA: Cancer
C18-20ICD-10: Codes for colorectal cancer
SD: Standard deviation
IQR: Interquartile range
N/A: Not applicable
OS: Overall survival
DFS: Disease-free survival
PFS: Progression-free survival
CT: Chemotherapy
RT: Radiotherapy
CRT: Chemoradiotherapy
BMI: Body mass index
ECOG: Eastern cooperative oncology group (performance status)
K-M: Kaplan-meier (survival analysis)
HR: Hazard ratio
CI: Confidence interval
SE: Standard error
* p*-value: Probability value
* N*: Number (Sample Size)

## References

[1] Sung H, Ferlay J, Siegel RL, Laversanne M, Soerjomataram I, Jemal A, Bray F. Global cancer statistics 2020: GLOBOCAN estimates of incidence and mortality worldwide for 36 cancers in 185 countries. CA Cancer J Clin. 2021;71(3):209–49. 33538338 10.3322/caac.21660

[2] Morgan E, Arnold M, Gini A, Lorenzoni V, Cabasag CJ, Laversanne M, Vignat J, Ferlay J, Murphy N, Bray F. Global burden of colorectal cancer in 2020 and 2040: incidence and mortality estimates from GLOBOCAN. Gut. 2023;72(2):338–44. 36604116 10.1136/gutjnl-2022-327736

[3] O’Sullivan B, Brierley J, Byrd D, Bosman F, Kehoe S, Kossary C, Piñeros M, Van Eycken E, Weir HK, Gospodarowicz M. The TNM classification of malignant tumours-towards common Understanding and reasonable expectations. Lancet Oncol. 2017;18(7):849–51. 28677562 10.1016/S1470-2045(17)30438-2 PMC5851445

[4] Eng C, Yoshino T, Ruíz-García E, Mostafa N, Cann CG, O’Brian B, Benny A, Perez RO, Cremolini C. Colorectal cancer. Lancet. 2024;404(10449):294–310. 38909621 10.1016/S0140-6736(24)00360-X

[5] Boakye D, Walter V, Jansen L, Martens UM, Chang-Claude J, Hoffmeister M, Brenner H. Magnitude of the Age-Advancement effect of comorbidities in colorectal cancer prognosis. J Natl Compr Canc Netw. 2020;18(1):59–68. 31910379 10.6004/jnccn.2019.7346

[6] Read WL, Tierney RM, Page NC, Costas I, Govindan R, Spitznagel EL, Piccirillo JF. Differential prognostic impact of comorbidity. J Clin Oncol. 2004;22(15):3099–103. 15284260 10.1200/JCO.2004.08.040

[7] Boakye D, Rillmann B, Walter V, Jansen L, Hoffmeister M, Brenner H. Impact of comorbidity and frailty on prognosis in colorectal cancer patients: A systematic review and meta-analysis. Cancer Treat Rev. 2018;64:30–9. 29459248 10.1016/j.ctrv.2018.02.003

[8] Kim HK, Song SO, Noh J, Jeong IK, Lee BW. Data configuration and publication trends for the Korean National health insurance and health insurance review & assessment database. Diabetes Metab J. 2020;44(5):671–8. 33115211 10.4093/dmj.2020.0207 PMC7643590

[9] Merchant SJ, Nanji S, Brennan K, Karim S, Patel SV, Biagi JJ, Booth CM. Management of stage III colon cancer in the elderly: practice patterns and outcomes in the general population. Cancer. 2017;123(15):2840–9. 28346663 10.1002/cncr.30691

[10] Nöpel-Dünnebacke S, Jütte H, Denz R, Feder IS, Kraeft AL, Lugnier C, Teschendorf C, Collette D, Böhner H, Engel L, et al. Causes of mortality in elderly UICC stage III colon cancer (CC) patients–Tumor-related death and competing risks from the German AIO colorectal study group Colopredict plus (CPP) registry. Cancer Med. 2022;11(8):1735–44. 35146948 10.1002/cam4.4540 PMC9041084

[11] Baretti M, Rimassa L, Personeni N, Giordano L, Tronconi MC, Pressiani T, Bozzarelli S, Santoro A. Effect of comorbidities in stage II/III colorectal cancer patients treated with surgery and neoadjuvant/adjuvant chemotherapy: A Single-Center, observational study. Clin Colorectal Cancer. 2018;17(3):e489–98. 29650416 10.1016/j.clcc.2018.03.010

[12] Lu CS, Chang PY, Chen YG, Chen JH, Wu YY, Ho CL. Stage III colon cancer: the individualized strategy of adjuvant chemotherapy for aged under and over 70. PLoS ONE. 2015;10(9):e0138632. 26382962 10.1371/journal.pone.0138632 PMC4575165

[13] Cai M, Gao Z, Liao J, Jiang Y, He Y. Frailty affects prognosis in patients with colorectal cancer: A systematic review and meta-analysis. Front Oncol. 2022;12:1017183. 36408138 10.3389/fonc.2022.1017183 PMC9669723

[14] McCleary NJ, Meyerhardt JA, Green E, Yothers G, de Gramont A, Van Cutsem E, O’Connell M, Twelves CJ, Saltz LB, Haller DG, et al. Impact of age on the efficacy of newer adjuvant therapies in patients with stage II/III colon cancer: findings from the ACCENT database. J Clin Oncol. 2013;31(20):2600–6. 23733765 10.1200/JCO.2013.49.6638 PMC3699725

[15] Sanoff HK, Carpenter WR, Stürmer T, Goldberg RM, Martin CF, Fine JP, McCleary NJ, Meyerhardt JA, Niland J, Kahn KL, et al. Effect of adjuvant chemotherapy on survival of patients with stage III colon cancer diagnosed after age 75 years. J Clin Oncol. 2012;30(21):2624–34. 22665536 10.1200/JCO.2011.41.1140 PMC3412313

